# Monodispersed AgNPs Synthesized from the Nanofactories of *Theobroma cacao* (Cocoa) Leaves and Pod Husk and Their Antimicrobial Activity

**DOI:** 10.1155/2022/4106558

**Published:** 2022-02-02

**Authors:** Johnson Kwame Efavi, Emmanuel Nyankson, Kwaku Kyeremeh, Gloria Pokuaa Manu, Kingsford Asare, Nathaniel Yeboah

**Affiliations:** ^1^College of Basic and Applied Sciences, Department of Materials Science & Engineering, University of Ghana, Accra, Ghana; ^2^College of Basic and Applied Sciences, Department of Chemistry, University of Ghana, Accra, Ghana

## Abstract

Silver nanoparticles (AgNPs) have been synthesized from the more chemically rich and diverse cocoa pod; the synthesis of silver nanoparticles from cocoa leaves, which are less rich and have low diversity in bioactive molecules, is yet to be achieved. In this work, AgNPs produced using the extracts of the cocoa leaf (CL) and cocoa pods (CP) have been investigated and their antimicrobial activity against *E. coli* was evaluated. UV-visible absorption spectroscopy was used to examine the reduction of silver ions in solution and the surface plasmon resonance of AgNPs. Scanning electron microscopy (SEM), energy-dispersive X-ray spectroscopy (EDX), dynamic light scattering (DLS), X-ray diffraction (XRD), and Fourier transform infrared spectroscopy (FTIR) were used to further characterize the nanoparticles. The crystalline nature of AgNPs was confirmed by XRD, and the purity and presence of elemental silver were determined by EDX. CL-AgNPs were observed to have a surface plasmon resonance of 425 nm, while CP-AgNPs had a surface plasmon resonance of 440 nm. CL-AgNPs had a significantly higher purity than CP-AgNPs. With a shorter nucleation time, the intensity of the UV-Vis spectrum was always higher in the case of CL-AgNPs, indicating a larger population of bioactive molecules available for CL-AgNPs synthesis. FTIR confirmed the presence of phenolic compounds in the leaf and pod extract, implying that water-soluble polyphenolic and flavonoid chemicals are responsible for nanoparticle reduction, capping, and stability. AgNPs generated from CL and CP extracts are polydispersed, with particle sizes of 10–110 nm and 20–680 nm, respectively, according to DLS. The corresponding zeta potentials measured are −2.7 mV for CL-AgNPs and −0.93 mV for CP-AgNPs. The zeta potential values suggest that the particles have long-term stability. Furthermore, CL-AgNPs outperformed CP-AgNPs in terms of antibacterial activity against *Escherichia coli*. CL-AgNPs were found to have a maximal inhibitory zone of 21 mm.

## 1. Introduction

The exploitation of quantum confinements and increased surface area [1] at the nanometer materials regime are at the forefront of the current and next-generation technologies to solve some of the pressing needs of mankind in the area of energy, environment, food security, medicine, shelter, water, and cybercrime [2].

Metallic and metal composite nanoparticles, among several kinds of nanomaterials, have intrigued scientists for over a century and are now widely used in biomedical sciences and engineering. The efficacy of these nanometer materials is size-, shape-, and concentration-dependent, making them appealing for biomedical applications [3]. These materials can be synthesized and modified with a variety of chemical functional groups, allowing them to be covalently linked with ligands, antibodies, and drugs of interest, opening up a wide range of potential applications in magnetic separation, biotechnology, and preconcentration of target analytes, targeted drug delivery, and vehicles for gene and more importantly diagnostic applications [4, 5]. For example, the antibacterial activity of various metal nanoparticles, such as silver colloids, is inversely proportional to their size; the smaller the silver nuclei, the greater the antibacterial activity. Furthermore, these nanoparticles' catalytic activity is influenced by their size, structure, shape, size distribution, and chemical-physical environment. As a result, maintaining control over the size and size distribution is critical.

Electrochemical techniques, chemical reduction and photochemical reduction, plasma arcing, ball milling, spray pyrolysis, ultrathin films, thermal evaporate, pulsed laser desorption, lithographic techniques, sputter deposition, layer-by-layer growth, diffusion flame methods, molecular beam epistaxis, and other physical and chemical methods can all be used to synthesize and stabilize metal nanoparticles [6, 7]. The experimental conditions, the kinetics of metal ions interaction with chemical reducing agents, and the adsorption processes of stabilizing agents have all been shown to have a significant impact on the size, texture, stability, and properties of chemically/physically synthesized metal nanoparticles [8, 9]. Furthermore, because current nanoparticle processing methods are costly, the development of a synthesis approach that allows for precise control of size, shape, stability, and characteristics has been a major focus.

Synthesis of metal nanoparticles from biological sources (plant and microbe sources) has become a research area of interest [8, 10]. These plants and microorganisms have bioactive compounds that can be used as a reducing and stabilizing agent in one-pot nanoparticle production. However, due to the difficulty in maintaining microbial cultures [10] and the advantages plants provide in terms of resource availability, security, reaction rate and convenience, and feasibility of the large-scale production, the synthesis of nanoparticles from plant extract is preferred.

Numerous studies have been published on the utilization of plant extracts in the manufacture of noble metal nanoparticles, particularly silver nanoparticles (AgNPs). This interest in plant extract methods stems from the ease of processing, low cost, and environmentally friendly procedures that produce nanoparticles with unique properties that can be used in biomedicine, fiber technology, electronics, food preservation, cosmetics, and other fields [11–13].

The significance of landforms and rock types in establishing unique regional distributions of plant ecosystems and promoting evolutionary diversification is influenced by the geology of a given location [14]. It is also known that ecosystems and habitats have a complicated interaction between physical and biological components, implying that microorganisms responsible for the reduction and stabilization of AgNPs during synthesis may differ depending on soil type and environment.

The geological settings in Ghana are unique and different from places where AgNPs have already been synthesized from plant parts; therefore, the bioactive molecular make-up of plant parts in such regions is expected to differ. Therefore, we hypothesize that the plant parts of cocoa in Ghana have different concentrations or proportions of bioactive molecule profiles and will thus produce different characteristics of nanoparticles that can be exploited in diverse biomedical applications. In this work, AgNPs have been synthesized from the extract of the cocoa leaf (CL) and cocoa pod (CP) and their characteristic features evaluated using UV-Vis, XRD, FTIR, EDX/SEM, and DLS. The biological activities of the nanoparticles have also been observed using *E. coli* in a disc diffusion method for antimicrobial testing.

## 2. Materials and Methods

### 2.1. Materials

Silver nitrate was procured from Sigma-Aldrich of UK. Fresh *Theobroma cacao* (Cocoa) fruits and leaves (see Figure S1 in the Supplementary Material) were harvested from a farm in Akropong, Eastern Region, Ghana, and transported to the laboratory for further processing.

### 2.2. Processing of Cocoa Husk Pod and Leaves into Liquid Extract

The cocoa pods and leaves were first washed and rinsed to remove any undesired foreign substances. To extract the beans from the pod, the cocoa pod was sliced and opened. The pod was chopped into pieces and air-dried for two weeks at room temperature. The leaves were also air-dried for one week. The dried pod and leaves were then ground in a Philips electric blender to obtain fine powder following a similar standard method in other works [15]. About 15 g of the powdered (cocoa pod and cocoa leave powder) samples was weighed and transferred into beakers containing ca.100 mL of distilled water. The mixture was boiled for about 10 minutes and cooled to ca. 25°C. The filtrate extract was collected after the mixture was filtered twice with Whatman No.1 filter sheets. The fresh plant extracts were stored in a refrigerator in 250 mL conical flasks and used for further experiments within 24 hours.

### 2.3. Green Synthesis of Cocoa Pod and Cocoa Leaf AgNPs

A 1 mM aqueous solution of silver nitrate (AgNO_3_) was prepared for each plant extract obtained by measuring a calculated mass of silver nitrate salts into a beaker. Distilled water from a wash bottle was then added and stirred continuously until it dissolved. The solution was then transferred to a 250 mL volumetric flask and diluted with more distilled water. In an Erlenmeyer flask, 200 mL of 1 mM silver nitrate was measured, and 20 mL of plant extract was added to the contents of the flask. The solution was then monitored for colour change characteristic of silver nanoparticle formation (reddish-brown, dark brown) caused by excitation of surface plasmon vibrations [16].

### 2.4. Characterization of AgNPs: UV-Vis, XRD, FTIR, SEM, EDX, and DLS

UV-Vis spectrophotometry was used to monitor the completion of silver ion bioreduction. That is, 1 mL samples of silver nitrate and plant extract solution mixture were taken at regular intervals, diluted with 2 mL deionized water, and absorbance was measured using UV-Vis spectrophotometry while looking for a characteristic peak of silver nanoparticles. In a GENESYS 10S UV-Vis (version v4.005 2L5S048209) scanner, the sample was scanned between 200 and 700 nm wavelengths. Powder X-ray Diffractometer (XRD) patterns recorded using an Empyrean PANalytical series 2 XRD with CuK*α* (1.54 Ǻ) radiation source and a tube running at 40 mA and 40 kV were used to determine the phase purity and crystallinity of AgNPs. X'Pert Highscore plus database software was used to identify the phases present in the samples. Energy-dispersive X-ray spectroscopy (EDX)-Zeiss EVO LS10 scanning electron microscopy (SEM) with Oxford INCA X-act detector was used to evaluate structural morphology and empirical elemental compositions, chemical purity, and stoichiometry. Using a Malvern Instrument ZEN 3600 Zetasizer Nano-ZX, the AgNPs were further characterized using dynamic light scattering (DLS) and zeta potential to quantify the volume-weighted hydrodynamic size and zeta potential, respectively. The measurement temperature was kept constant at 25°C.

### 2.5. Antibacterial Activity Test

Silver nanoparticles made from cocoa leaves and those made from cocoa pods were tested for antibacterial activity against *Escherichia coli* (*E. coli*). The bacteria for this test were obtained from the University of Ghana's Noguchi Memorial Institute utilizing the disc diffusion methods outlined in [17].

Small disks cut out from filter paper were soaked in silver nanoparticle solutions of the cocoa extracts. Filter papers were also soaked in distilled water, aqueous silver nitrate, and plant extracts to serve as controls. The filter papers were then air-dried. Plates containing *E. coli* inoculum mixed with nutrient agar were then prepared. The plates were then partitioned for placements of filter paper. On one type of plate, 4 filter papers from distilled water treatment, AgNPs from cocoa leaves, and pod and AgNPs from chemical synthesis were placed. On another type of plate (control plate), filter paper treatments of distilled water, cocoa plant extracts, and aqueous silver nitrate treatments were placed. In a growth chamber at 37°C, the plates were incubated for 48 hours. For each type of filter paper treatment, the zones of inhibition established after 48 hours were evaluated.

## 3. Results and Discussion

Studies have shown that metal nanoparticles have a strong absorption band and produce a distinctive colloidal suspension colour due to surface plasmon resonance (SPR) [18]. The reaction of aqueous extracts of cocoa leaves and pods with an aqueous solution of silver nitrate resulted in the bioreduction of silver ions to silver. The extracts changed colour from pale yellow to dark brown (see Figures S2 (a and b, respectively) in the Supplementary material), showing the production of silver nanoparticles in the colloidal suspension. This colour change in the suspension confirms the formation of AgNPs as a consequence of surface plasmon vibrations of AgNPs being excited [18, 19].

**Figure 1:**
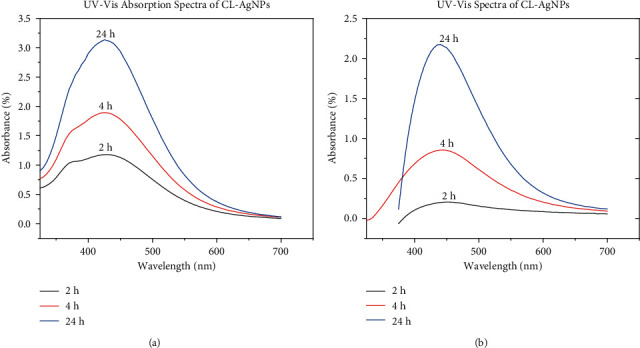
(a) UV-Vis absorption spectra of CL-AgNPs. (b) UV-vis absorption spectra of CP-AgNPs.

The formation of AgNPs from both cocoa leaf and cocoa pod extracts was confirmed using UV-Vis spectroscopy at absorbance peak wavelengths of 425 nm and 440 nm, respectively. This is consistent with the range at which absorbance peaks of silver nanoparticles occur [19].  Figure 1(a) shows the absorption spectra of silver nanoparticles formed from cocoa leaves broth, and Figure 1(b) shows cocoa pod broth. It can be observed that in both cases, there was a steady increase in intensity with reaction time.

Because the SPR band intensity and wavelength are affected by factors that influence the electron charge density on the particle surface, such as metal type and structure, particle size and shape, composition, and the dielectric constant of the surrounding medium, as theoretically described by Mie theory, the difference in the observed absorption peak is expected [20, 21]. This suggests that the kinetics of the CL-AgNPs and CP-AgNPs formation and the number of nanoparticles produced are different. It is also observed that the absorption peaks did not shift to different wavelengths as the reaction time changed for both leaves and cocoa pods, demonstrating that the as-synthesized silver nanoparticles are uniformly distributed and stable with the same properties [21].

**Figure 2:**
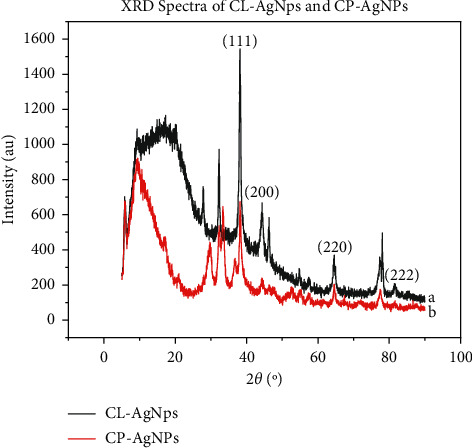
(a) XRD spectra of CL-AgNPs. (b) XRD spectra of CP-AgNPs.

XRD analysis was used to examine the phases present in the synthesized CL-AgNPs and CP-AgNPs.  Figure 2 shows the patterns of AgNPs formed from the broth of cocoa leaves and pods superimposed. Peaks were observed at 2*θ* = 38.18°, 42.32°, 64.43°, 77.44°, and 81.52° for both CL-AgNPs and CP-AgNPs. These peaks are attributed to crystallographic planes (111), (200), (220), (311), and (222), which correspond to characteristic Bragg reflections in silver [22]. The observance of characteristic Bragg diffraction peaks in the silver nanoparticles from both sources of extract confirms the crystalline morphology of the nanoparticles. There is no difference in the crystallographic planes responsible for the observed diffractions except that the intensity of the spectra of CL-AgNPs is higher, indicating increased levels of crystallinity.

It is reported that during the production of nanoparticles from nanofactories of plant extract, AgNO_3_ dissociate into Ag⁺ and NO^3‾^ in the aqueous medium and that the plant extracts contain a high level of bioactive molecules that acts as both reducing and stabilizing agents. The plant extract releases bioactive compounds, which react with the aqueous AgNO_3_ solution, forming a bioactive substrate complex as Ag^+^ ions join with the bioactive molecules. The functional groups released by the bioactive molecules interact with the silver ions, resulting in the formation of AgNP. The silver nanoparticle then reacts with proteins produced by the plant extract, resulting in protein-capped silver nanoparticles [21].

**Figure 3:**
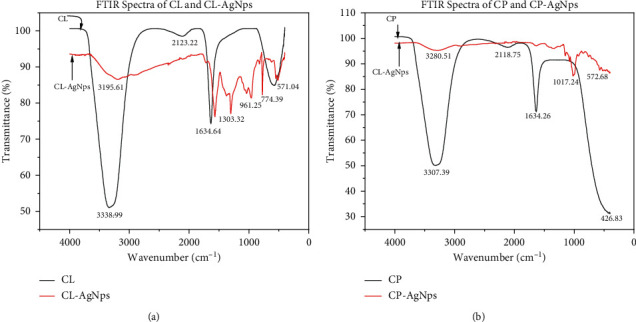
(a) FTIR spectra of Cl and CL-AgNPs. (b) FTIR spectra of CP and CP-AgNPs.

Plant extracts and AgNPs generated were analyzed using Fourier transform infrared spectroscopy (FTIR) to detect the functional groups present that could be responsible for the nanoparticles' reduction and efficient capping and stability. Figures 3(a) and 3(b) show the superimposition of FTIR spectra of CL extract and the CL-AgNP spectra and that of CP extract and the corresponding CP-AgNPs, respectively. The spectra reveal strong absorption bands at 3338, 2123, 1634, and 571 cm^−1^. For CL extract, the 3338 cm^−1^ band is associated with hydroxyl (OH) groups, which consist of alcohol and phenolic compounds with hydroxyl bonds [23]. The 2123 cm^−1^ band is attributed to triple bond monosubstituted alkyne [23]. Due to aromatic ring deformation, the 1634 cm^−1^ band is connected with stretching vibrations of C=C groups [24]. Aliphatic iodo compounds (C-I) stretch is attributed to the band at 571 cm^−1^ [25]. The CL-AgNPs spectra show similar bands (3338, 2123, 1634, and 571 cm^−1^) but with a shift towards a lower wavelength, as seen in Figure 3(a). This shows that the biomolecules in the extract and the AgNPs formed have a greater interaction [26]. The binding of C=C groups with AgNPs is responsible for the shift observed at 1634 cm^−1^. The emergence of phenolic and flavonoid functional groups in the spectra of CL-AgNPs and the fact that band shifts and intensity decrease suggest that they were actively involved in the bioreduction and stability of CL-AgNPs. This corroborates a number of phytochemical studies using cocoa plant, which showed that in cocoa, the main bioactive molecules are phenols or phenolic compounds [27].

From Figure 3(b), bands are assigned at 3307, 2118, and 1634 cm^−1^ for both the CP extract and CP-AgNPs produced. However, the bands are not as strong as those observed from the CL extract. This could be owing to the fact that they contain fewer polyphenol chemicals, which have been linked to nanoparticle reduction and stabilization [28]. These bands 3307, 2118, and 1634 cm^−1^, as discussed earlier, are assigned to alcohols/phenolic compounds (-OH), triple bond monosubstituted alkyne, and stretching vibrations of C=C groups, respectively. The appearance of the 1017 cm^−1^ band on the CP-AgNPs spectra is associated with triple bond alkynes [23].

The near disappearance of these bands after the bioreduction is indicative of the fact that polyphenol compounds are primarily responsible for the bioactivity AgNPs production [29]. Also, as seen in Figure 3(a), new bands also appear in the spectra of the AgNPs at 1303, 961, and 774 cm^−1^ wavenumbers. This observation suggests that their presence in the crude extract was overshadowed, causing them not to be detected [29].

The above assignment of spectra and description shows that the major chemical elements of cocoa, regardless of the plant parts, are a cocktail mixture of polyphenolics that include flavonoids. The IR spectra of crude cocoa leaves (CL) and pods (CP) do not disappoint, with smooth broad peaks at 3338 cm^−1^ and 3307 cm^−1^, respectively, due to the lack of any further peaks in the 1725–1700 cm^−1^ range, and are thus assigned to an alcohol O-H stretch vibration. The peaks at 1634 cm^−1^ in Figures 3(a) and 3(b) were too low in wavenumber to be assigned to a ketone; even for the instance of conjugation to an unsaturated group or if assigned to an aldehyde, the aldehydic hydrogen must be at 2900–2700 cm^−1^. The O-H stretches in the spectra must be substantially wider to assign these peaks at 1634 cm^−1^ to the C=O of carboxylic acid. As a result, the peak at 1634 cm^−1^ was unambiguously ascribed to a C=C stretch of an alkene or, more likely, an aromatic. Absorption peaks at 2123 cm^−1^ and 2118 cm^−1^ for both CL and CP are possibly aromatic overtone and combination bands representing most probably trisubstituted polyphenolic aromatic rings. In the IR spectrum of crude CL and CP extracts, peaks at 571 cm^−1^ and 426 cm^−1^ are indicative of residual brominated compounds like methyl bromide that form the major constituents of cocoa pesticides. The IR spectra of CL and CP lack fine structure due to the highly concentrated nature of these extracts.

The IR spectra of both CL-AgNPs and CP-AgNPs have a fine structure because the synthesized particles are normally purified and freed from the rest of the chemical components in the extracts. The O-H stretch vibration frequencies 3195 cm^−1^ and 3280 cm^−1^ detected in both the CL-AgNPs and CP-AgNPs, respectively, are much broader than those in the spectrum of the crude raw extracts. This is understandable since the broadness is indicative of the many geometric configurations that exist around the O-H bonds especially in the structure of the synthesized silver nanoparticles. The absorption peaks at 1303, 961, and 774 cm^−1^ in the fingerprint region of the IR spectrum of CL-AgNPs are difficult to assign unambiguously but are most likely related to the aromatics; these peaks have become characteristic of all the silver nanoparticles we have synthesized from CL. The same observation is also true for CP-AgNPs, where fingerprint absorption peaks are seen at 1000 and 1017 cm^−1^. However, small amounts of the residual brominated pesticides are still left even after purification of the synthesized nanoparticles. The reduction of silver ions in CL-AgNPs and CP-AgNPs appears to be caused by phenolic hydroxyl ligands.

Hence, we conclude that the silver (I) ions reduction during the synthesis of the nanoparticles is most probably facilitated by the polyphenolic natural products that are very characteristic of all cocoa extracts and known to be present even in refined cocoa products like chocolates, cocoa powders, and cocoa butter.

**Figure 4:**
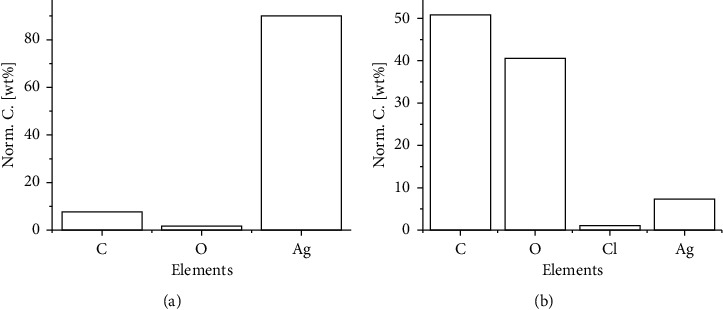
(a) EDX analysis of CL-AgNPs. (b) EDX analysis of CP-AgNPs.

EDX analysis of the AgNPs synthesized from both extracts of cocoa leaves and the cocoa pod is presented in Figure 4. Signals of silver were detected in the EDX along with other elements of O, C, and Cl, with percentage compositions showing the purity of the synthesized Ag nanoparticles. It is observed that there is a higher percentage of Ag content in the CL-AgNPs than in CP-AgNPs. The other signals present in the EDX may be coming from traces of compounds bound to the AgNPs. This goes to confirm that the bioactive molecules in cocoa leaves extract are more effective in reducing and stabilizing nanoparticles, resulting in higher purity of Ag for the same processing variables. The full EDX spectrum of CL-AgNPs and CP-AgNPs can be found in the Supplementary Materials (see Figures S5 and S6, respectively, in the Supplementary Materials).

**Figure 5:**
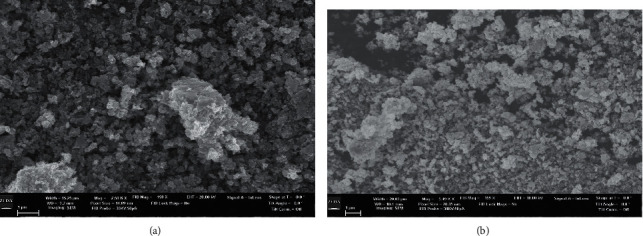
(a) SEM of CL-AgNPs. (b) SEM of CP-AgNPs.

The morphology of the CL-AgNPs and CP-AgNPs is shown in the SEM image in Figure 5. AgNP aggregates can be seen in both cases; however, they are not in direct contact even inside the aggregates, indicating that bioactive capping agents have stabilized the nanoparticles. It is observed in Figure 5(a) that the size of aggregation in CL-AgNPs is smaller than that of the CP-AgNPs depicted in Figure 5(b). By observation, CL-AgNPs have a greater and denser nanoparticle population than CP-AgNPs.

The size population distribution of the nanoparticles was analyzed using DLS techniques. It is a statistical analytical method that uses laser light scattered by Brownian motion nanoparticles in a colloidal suspension.  Figure S3 in Supplementary Materials shows the DLS pattern of silver nanoparticles in colloidal suspension synthesized using cocoa leaf extract (CL-AgNPs). The particle size distribution profile shows two peaks indicating polydispersion of the population (see Figure S3(a) in the Supplementary Materials). The particle size distribution is characterized by aggregates with size populations within 110 nm, having a very narrow scatter. The mean polydispersity index (PDI), a parameter for describing nanoparticle size distributions, gives information on many size populations observed at the same time. This is calculated based on zeta potential measurements and is 0.45 for the produced AgNPs. This value of polydispersity index (PDI) of the CL-AgNPs shows that the nanoparticles have a restricted size distribution and the technique used for the measurements is good [30]. In addition, statistically, the distribution profile in Figure S3a in Supplementary Materials suggests that about 95% of the distribution is within 2 standard deviations from the value of 100 nm and that 30% of them have sizes above ∼20 nm and about 22% have sizes of ∼55 nm.

The measured zeta potential of the particles in colloidal solution is also shown in Figure S3b in the Supplementary Materials. The movement of nanoparticles under the effect of an applied electric field and surface charges and the colloidal medium alter the zeta potential values [31]. The particles have a measured average negative zeta potential of −0.54 mV, indicating that they are highly stable due to electrostatic repulsive force between them [30].

The particles distribution profile of the CP-AgNPs is also presented in Figure S4a (Supplementary Material) with the corresponding zeta potential measurement in Figure S4b in Supplementary Materials. The particles are polydispersed with two overlapping size populations. The particles size distribution is characterized by a much larger aggregate with a size range below 680 nm compared to CL-AgNPs, having a very narrow scatter towards smaller sizes and a broad size scatter to larger particles. The average calculated PDI is 0.74, indicating a broader spread of the size distribution. The distribution further shows that 90% of particles have sizes below ∼700 nm. The particles have an average measured negative zeta potential value of −0.72 mV, also pointing to the fact that the particles are highly stable. The negative value validates the particles' repulsion and demonstrates their stability basically because they do not show any disposition to come together, thereby preventing agglomeration.

This difference in particle sizes and the spread of the distribution as observed in CL-AgNPs and CP-AgNPs is attributed to a higher population of bioactive molecules in the leaf extract participating in the reduction of Ag^+^, yielding more abrupt nucleation and faster growth of the nanoparticles, while producing small agglomerates [32].

**Figure 6:**
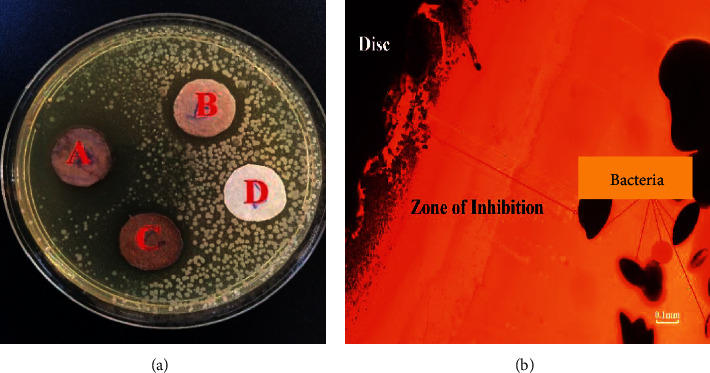
(a) Zone of exhibition of antimicrobial activity. (b) Optical microscope image of the zone of exhibition of CL-AgNPs.

Using the agar well diffusion assay, the antibacterial activity of biosynthesized silver nanoparticles, AgNO3, from cocoa leaf extract and the cocoa pod was investigated against Gram-negative (*E. coli*) bacteria, and the zone of inhibition was observed as shown in Figure 6. In the disk diffusion method, results were obtained by measuring the zone of inhibition after the incubation period.

The zones of inhibition after the incubation period were inspected and measured on filter paper discs with CL-AgNPs (A), CP-AgNPs (B), chemically synthesized silver nanoparticles (C), and distilled water as control (D). The chemically synthesized silver nanoparticles had an average particle size of 25–450 nm [33]. The antibacterial activity of the synthesized AgNPs against *E. coli* was excellent. As shown in Figure 6(a) (disc A), silver nanoparticles made from cocoa leaf extracts had the greatest inhibitory zone with a diameter of around 21 mm. This value of 21 mm (2.1 cm) is comparable to that of the measured zones when Ampicillin is used as a positive control in various investigations in our laboratory against strains of *E. coli*.

On the other hand, there was no zone of inhibition in the negative control (distilled water). These findings also imply that particle binding to bacteria is influenced by the surface area available for interaction. The highest zone of inhibition demonstrated by CL-AgNPs against CP-AgNPs suggests that the CL-AgNPs are of smaller sizes. Generally, small nanoparticles have a wider surface area for bacterium contact than larger particles, resulting in increased antibacterial activity.  Table 1 compares the microbial activity of the silver nanoparticles synthesized in this work to that of other published works. It can be seen that the zone of inhibition for *E. coli* for the standard antibiotics is 8 mm and that the green synthesized AgNPs are all below our synthesized CL-AgNPs, thereby confirming the effectiveness of the CL-AgNPs.

## 4. Conclusion

The characteristics of the respective nanoparticles (CL-AgNPs and CP-AgNPs) were evaluated using UV-Vis, XRD, FTIR, SEM, EDX, and DLS after AgNPs were effectively synthesized from the cocoa plant leaf and pod extracts. The phytochemicals of phenols in the plant extracts functioning as reducing, stabilizing, and capping agents are responsible for the effective synthesis of AgNPs from the extracts, as confirmed by FTIR analysis. CL-AgNPs were observed to have smaller particle sizes and higher purity in the 10–100 nm range. This indicates that the cocoa leaf extract has richer phenol compounds than the pod extract resulting in a different rate of nucleation and growth of the AgNPs. The importance of biological extracts as a nanofactory, with their intrinsic biomolecular reducing agent as a potential veritable instrument for nanoparticle synthesis, is highlighted by these findings. They also provide an analytical option for screening bioactive compounds from biological samples for nanoparticle synthesis. CL-AgNPS and CP-AgNPs both had antibacterial activity against *E. coli*, with CL-AgNPs having a zone of inhibition of 21 mm.

## Figures and Tables

**Table 1:** Zones of inhibition of green synthesized AgNPs against *E. coli*.

Sample	Zone of inhibition (mm)	Reference
CL-AgNps	21	This article
Pen-Strep (standard antibiotic)	8	[34]
GPP-AgNPs	1.8	
GPF-AgNPs	3.0	
RPP-AgNPs	2.5	
RPF-AgNPs	4.1	
*T. triangulare* AgNPs	2.55	[35]
50 *μ*g/ml AgNPs	12	[36]
100 *μ*g/ml AgNPs	15	
25 *μ*g/ml AgNPs	7	[37]
50 *μ*g/ml AgNPs	9	
100 *μ*g/ml AgNPs	10	
0.25 mM AgNPs	0	[38]
0.5 mM AgNPs	8.33	
1 mM AgNPs	16.67	

## Data Availability

All data generated or analyzed during this study are included in this published article and its supplementary information files.

## References

[1] Kodolov V. I., Zaikov G. E., Haghi A. (2016). *Applied Nanotechnology: Materials and Applications*.

[2] Akbari B., Tavandashti M. P., Zandrahimi M. (2011). Particle size characterization of nanoparticles–a practicalapproach. *Iranian Journal of Materials Science and Engineering*.

[3] Mayer K. M., Hafner J. H. (2011). Localized surface plasmon resonance sensors. *Chemical Reviews*.

[4] Meng X., Seton H. C., Lu L. T., Prior I. A., Thanh N. T. K., Song B. (2011). Magnetic CoPt nanoparticles as MRI contrast agent for transplanted neural stem cells detection. *Nanoscale*.

[5] Zhang H.-W., Liu Y., Sun S.-H. (2010). Synthesis and assembly of magnetic nanoparticles for information and energy storage applications. *Frontiers of Physics in China*.

[6] Zhang G., Wang D. (2008). Fabrication of heterogeneous binary arrays of nanoparticles via colloidal lithography. *Journal of the American Chemical Society*.

[7] Starowicz M., Stypuła B., Banaś J. (2006). Electrochemical synthesis of silver nanoparticles. *Electrochemistry Communications*.

[8] Dale L., Huber D. J. S. (2005). Synthesis, properties, and applications of iron nanoparticles. *Small*.

[9] Sanghi R., Verma P. (2009). Biomimetic synthesis and characterisation of protein capped silver nanoparticles. *Bioresource Technology*.

[10] Kalishwaralal K., Deepak V., Ram Kumar Pandian S. (2010). Biosynthesis of silver and gold nanoparticles using Brevibacterium casei. *Colloids and Surfaces B: Biointerfaces*.

[11] Popescu M. (2010). Biogenic production of nanoparticles. *Digest Journal of Nanomaterials & Biostructures*.

[12] Baruwati B., Polshettiwar V., Varma R. S. (2009). Glutathione promoted expeditious green synthesis of silver nanoparticles in water using microwaves. *Green Chemistry*.

[13] Rajput N. (2015). Methods of preparation of nanoparticles-a review. *International Journal of Advances in Engineering & Technology*.

[14] Kruckeberg A. R. (2004). *Geology and Plant Life: The Effects of Landforms and Rock Types on Plants*.

[15] Banerjee P., Satapathy M., Mukhopahayay A., Das P. (2014). Leaf extract mediated green synthesis of silver nanoparticles from widely available Indian plants: synthesis, characterization, antimicrobial property and toxicity analysis. *Bioresources and Bioprocessing*.

[16] Li S., Shen Y., Xie A. (2007). Green synthesis of silver nanoparticles using Capsicum annuum L. extract. *Green Chemistry*.

[17] Krause A., Cowles E. A., Gronowicz G. (2000). Integrin-mediated signaling in osteoblasts on titanium implant materials. *Journal of Biomedical Materials Research*.

[18] Nithya R., Ragunathan R. (2009). Synthesis of silver nanoparticle using Pleurotus sajor caju and its antimicrobial study. *Digest Journal of Nanomaterials and Biostructures*.

[19] Shankar S. S., Rai A., Ahmad A., Sastry M. (2004). Rapid synthesis of Au, Ag, and bimetallic Au core-Ag shell nanoparticles using Neem (Azadirachta indica) leaf broth. *Journal of Colloid and Interface Science*.

[20] Płaza G. A., Chojniak J., Banat I. M. (2014). Biosurfactant mediated biosynthesis of selected metallic nanoparticles. *International Journal of Molecular Sciences*.

[21] Jain P. K., Lee K. S., El-Sayed I. H., El-Sayed M. A. (2006). Calculated absorption and scattering properties of gold nanoparticles of different size, shape, and composition: applications in biological imaging and biomedicine. *The Journal of Physical Chemistry B*.

[22] Kratošová G., Vávra I., Horská K. (2013). Synthesis of metallic nanoparticles by diatoms and chrysophytes–prospects and applications. *Green Biosynthesis of Nanoparticles*.

[23] Drobniak A., Mastalerz M. (2006). Chemical evolution of Miocene wood: example from the Belchatow brown coal deposit, central Poland. *International Journal of Coal Geology*.

[24] Shankar S. S., Ahmad A., Sastry M. (2003). Geranium leaf assisted biosynthesis of silver nanoparticles. *Biotechnology Progress*.

[25] Coates J. (2000). *Interpretation of Infrared Spectra, a Practical Approach*.

[26] Tripathy A., Raichur A. M., Chandrasekaran N., Prathna T. C., Mukherjee A. (2010). Process variables in biomimetic synthesis of silver nanoparticles by aqueous extract of Azadirachta indica (Neem) leaves. *Journal of Nanoparticle Research*.

[27] Vriesmann L. C., de Mello Castanho Amboni R. D., de Oliveira Petkowicz C. L. (2011). Cacao pod husks (Theobroma cacao L.): composition and hot-water-soluble pectins. *Industrial Crops and Products*.

[28] Vijaymohan K., Kamala N. S., Udayaprakash N., Madhankumar D. (2012). One step green synthesis of silver nanoparticles using extracts of T. amni and P. som-niferum. *Colloids and Surfaces B: Biointerfaces*.

[29] Li Q., Mahendra S., Lyon D. Y. (2008). Antimicrobial nanomaterials for water disinfection and microbial control: potential applications and implications. *Water Research*.

[30] Kumar C. G., Mamidyala S. K., Reddy M. N., Reddy B. V. S. (2012). Silver glyconanoparticles functionalized with sugars of sweet sorghum syrup as an antimicrobial agent. *Process Biochemistry*.

[31] Pecora R. J. (2000). Dynamic light scattering measurement of nanometer particles in liquids. *Journal of Nanoparticle Research*.

[32] Tanko Y., Kamba B., Saleh M. I., Musa K. Y., Mohammed A. (2008). Anti-nociceptive and anti-inflammatory activities of ethanolic flower extract of Newbouldia laevis in mice and rats. *International Journal of Applied Research in Natural Products*.

[33] Iravani S., Korbekandi H., Mirmohammadi S. V., Zolfaghari B. (2014). Synthesis of silver nanoparticles: chemical, physical and biological methods. *Research in Pharmaceutical Sciences*.

[34] Simon S., Sibuyi N. R. S., Fadaka A. O., Meyer M., Madiehe A. M., du Preez M. G. (2021). The antimicrobial activity of biogenic silver nanoparticles synthesized from extracts of red and green European pear cultivars. *Artificial Cells, Nanomedicine, and Biotechnology*.

[35] Ojo O. A., Oyinloye B. E., Ojo A. B. (2017). Green synthesis of silver nanoparticles (AgNPs) using Talinum triangulare (Jacq.) Willd. leaf extract and monitoring their antimicrobial activity. *Journal of Bionanoscience*.

[36] Sharma S., Kumar S., Bulchandini B., Taneja S., Banyal S. (2013). Green synthesis of silver nanoparticles and their antimicrobial activity against gram positive and gram negative bacteria. *International Journal of Biotechnology and Bioengineering Research.*.

[37] Patil S., Sivaraj R., Rajiv P., Venckatesh R., Seenivasan R. (2015). Green synthesis of silver nanoparticle from leaf extract of Aegle marmelos and evaluation of its antibacterial activity. *International Journal of Pharmacy and Pharmaceutical Sciences*.

[38] Vanlalveni C., Rajkumari K., Biswas A., Adhikari P. P., Lalfakzuala R., Rokhum L. (2018). Green synthesis of silver nanoparticles using Nostoc linckia and its antimicrobial activity: a novel biological approach. *BioNanoScience*.

