# Supplementary material for: Monodispersed AgNPs Synthesized from the Nanofactories of Theobroma cacao (Cocoa) Leaves and Pod Husk and Their Antimicrobial Activity
Source: Int J Biomater. 2022 Feb 2;2022:4106558. doi: 10.1155/2022/4106558 (PMC8828336; doi:10.1155/2022/4106558)
Supplement: Supplementary Materials — on the list of figures are provided in a separate file named “COCOA'S SUPPLEMENTARY INFORMATION.” [file 4106558.f1.docx]

**SUPPLEMENTARY INFORMATION**

Monodispersed AgNPs Synthesised from the Nanofactories of Theobroma Cacao (Cocoa) Leaves and Pod Husk and their Antimicrobial Activity

Johnson Kwame Efavi­­­^1a^, Emmanuel Nyankson^1a*^, Kwaku Kyeremeh^1b^, Gloria Pokuaa Manu^1a^, Kingsford Asare^1a^, Nathaniel Yeboah^1a^

*College of Basic and Applied Sciences (Departments of Materials Science & Engineering^1a^ and Chemistry^1b^)*

*^1^University of Ghana*

*Accra, Ghana*

*Corresponding Author Email: jkefavi@ug.edu.gh*

**List of Figures:**


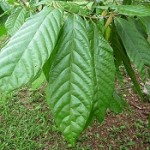

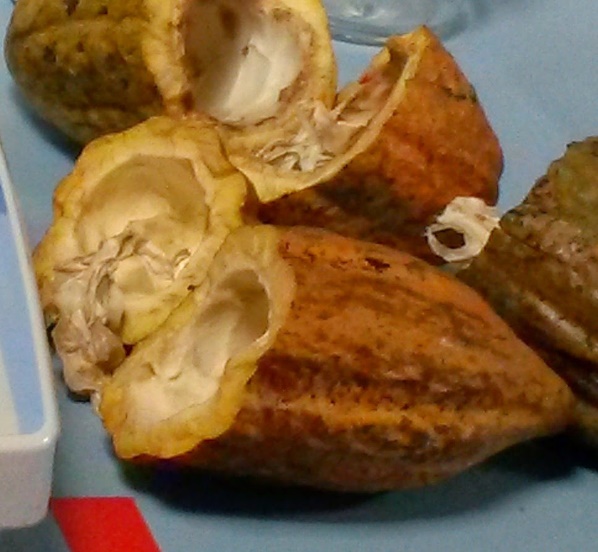


**a**

**b**

Figure S1: (a) Image of Cocoa Leaf (b) Image of Cocoa Pod


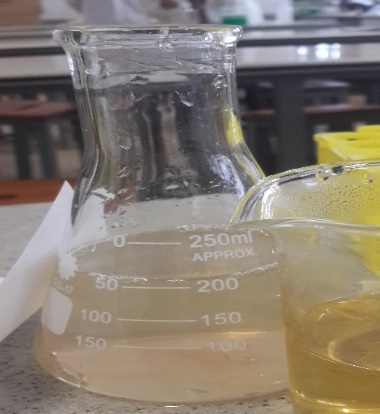

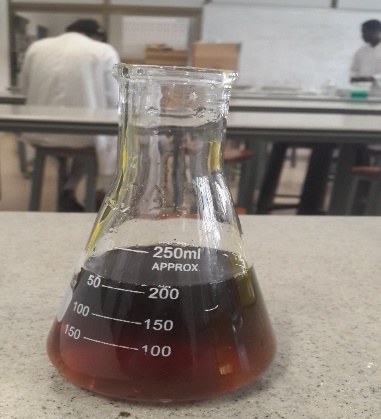


Figure S2: (a) Cocoa Extract (b) Colloidal Suspension of Silver Nanoparticles


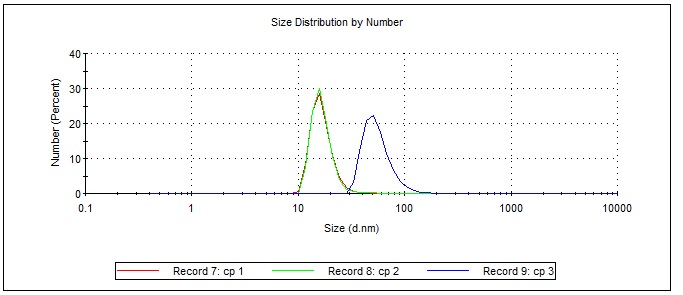

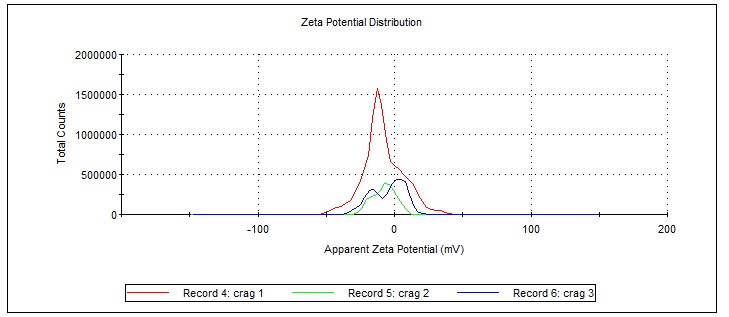


Figure S3: (a) DLS Analysis CL-AgNPs (b) ZETA Potential of Measurement CL-AgNPs


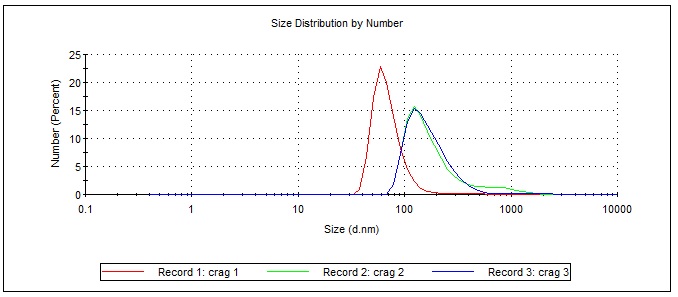

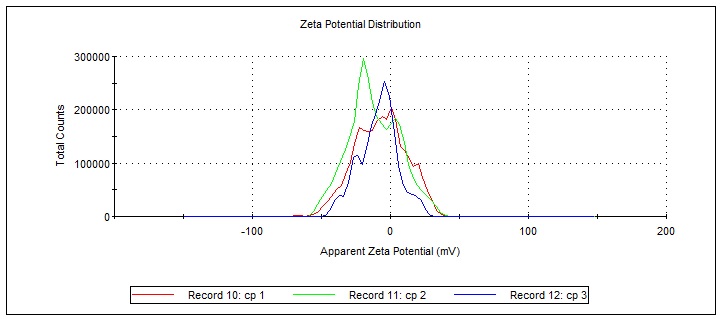


Figure S4: (a) DLS Analysis CP-AgNPs (b) ZETA Potential of Measurement CP-AgNPs


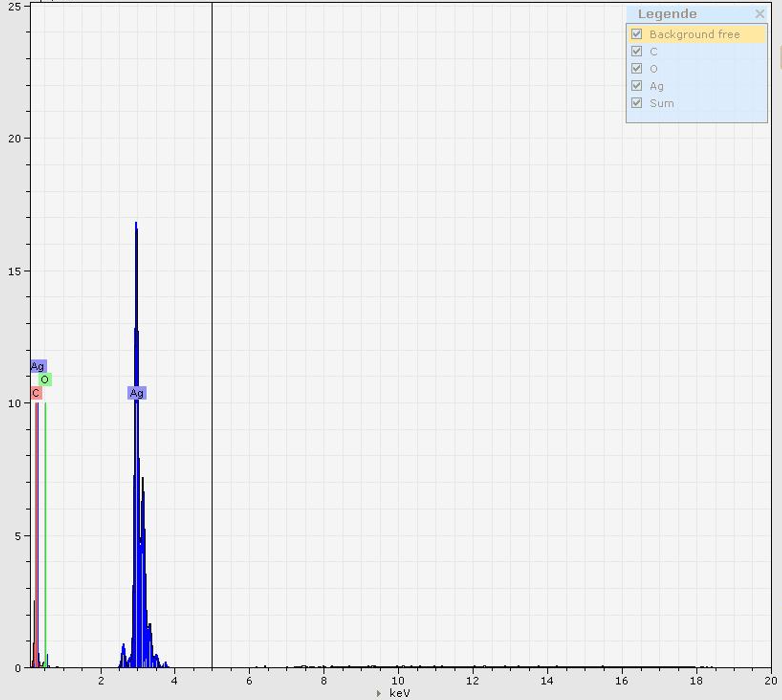


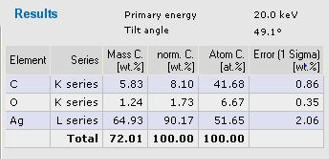


(b)

(a)


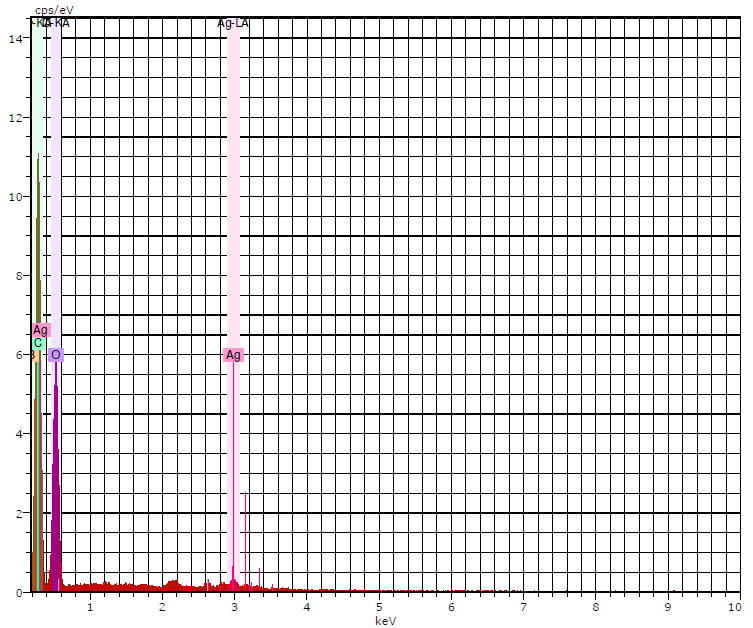
Figure S5: EDX spectrum and its elemental composition of CL – AgNPs ((a) and (b) respectively)


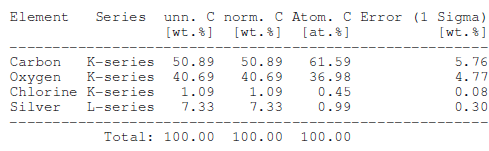


(b)

(a)

Figure S6: EDX spectrum and its elemental composition of CP – AgNPs ((a) and (b) respectively)
